# Supplementary figures and images for: Secondary structure of the human mitochondrial genome affects formation of deletions
Source: BMC Biol. 2023 May 8;21:103. doi: 10.1186/s12915-023-01606-1 (PMC10166460; doi:10.1186/s12915-023-01606-1)

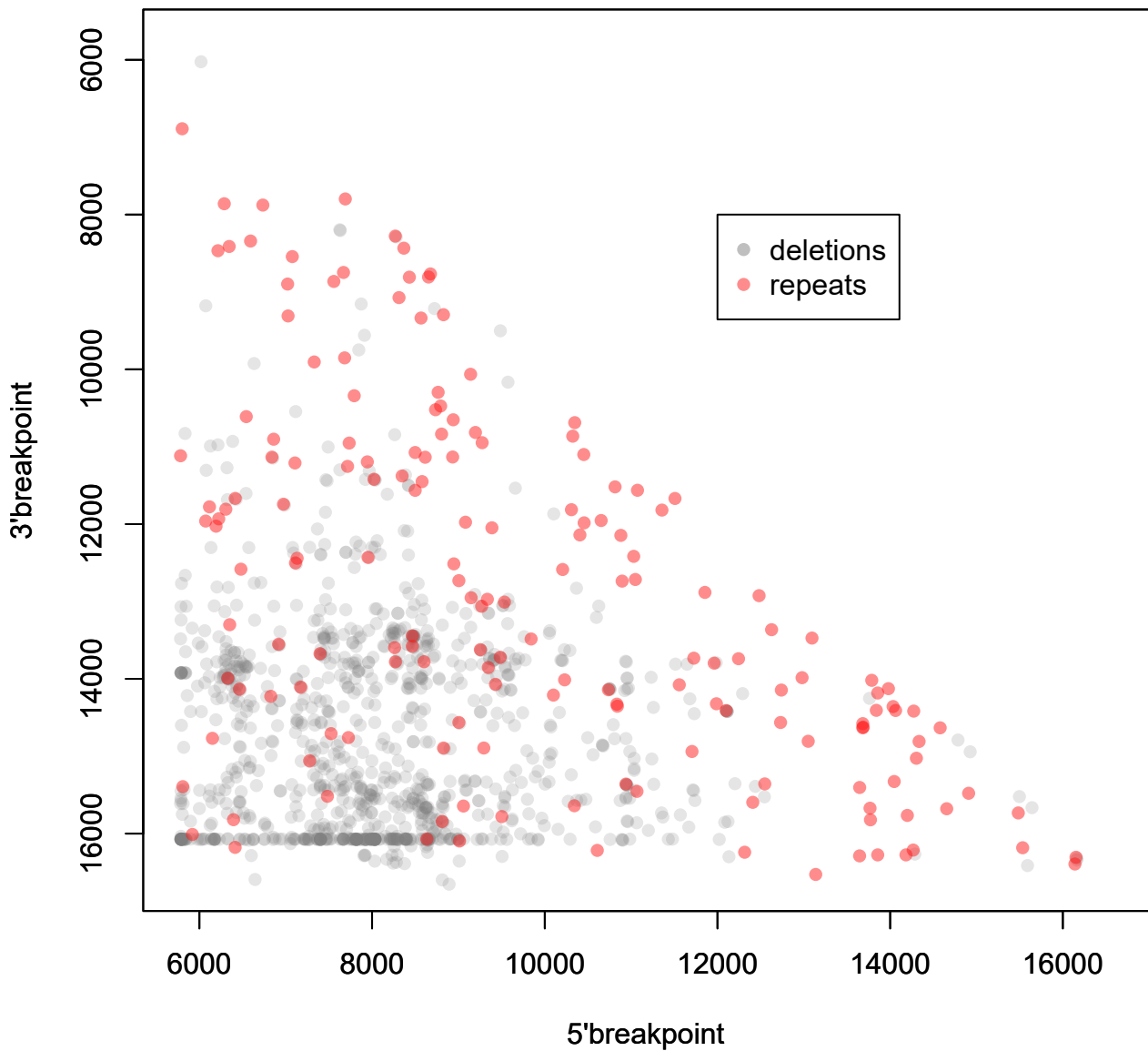

Supplement: Supplementary file 1 — Additional file 1. Distribution of the perfect direct repeatsand deletions from MitoBreakin the major arc. [file 12915_2023_1606_MOESM1_ESM.pdf]

end of breakpoint

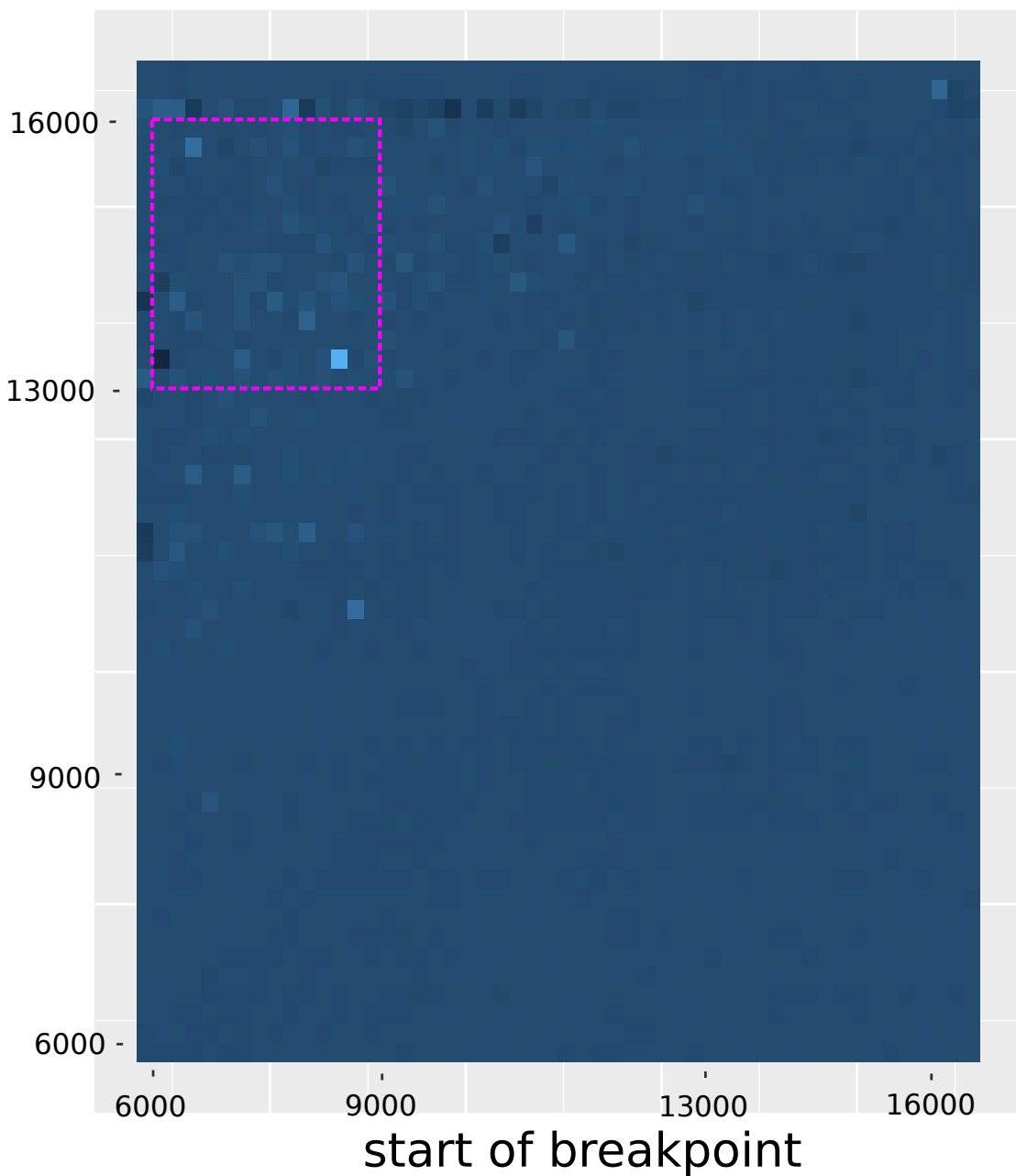

Supplement: Supplementary file 2 — Additional file 2. The third principal component scores, associated with aging-related deletions of healthy samples from a paper [36]. The contact, marked by the pink square, is characterized by the increased scores. [file 12915_2023_1606_MOESM2_ESM.pdf]

# mtDNA Hi-C contact matrix

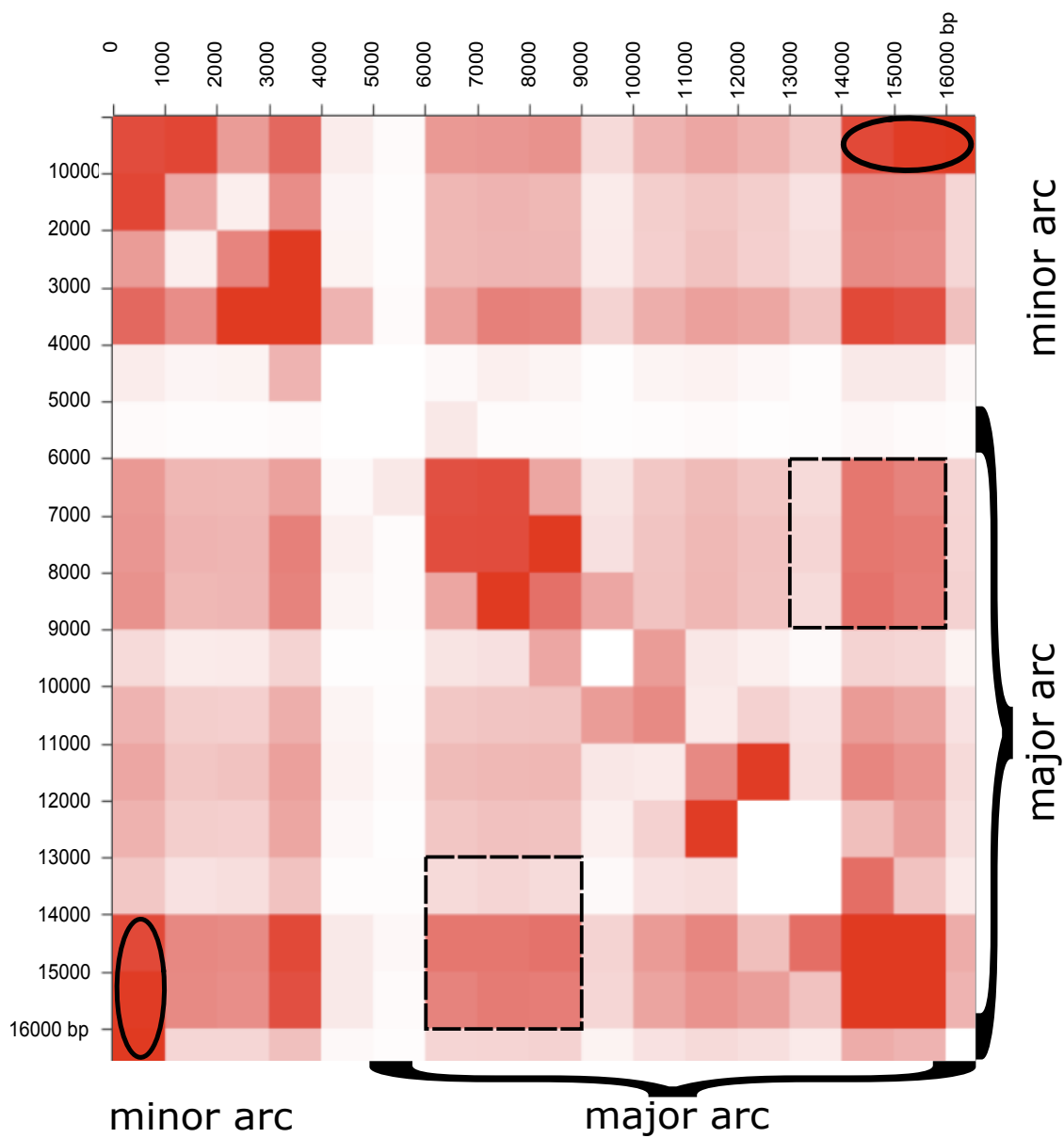

Supplement: Supplementary file 3 — Additional file 3. Hi-C contact matrix of mtDNA obtained from the human lymphoblastoid cells. Dotted squares mark the potential contact zones. Ovals mark the contacts, emphasizing the circularity of mtDNA. [file 12915_2023_1606_MOESM3_ESM.pdf]

# mtDNA Hi-C contact matrix

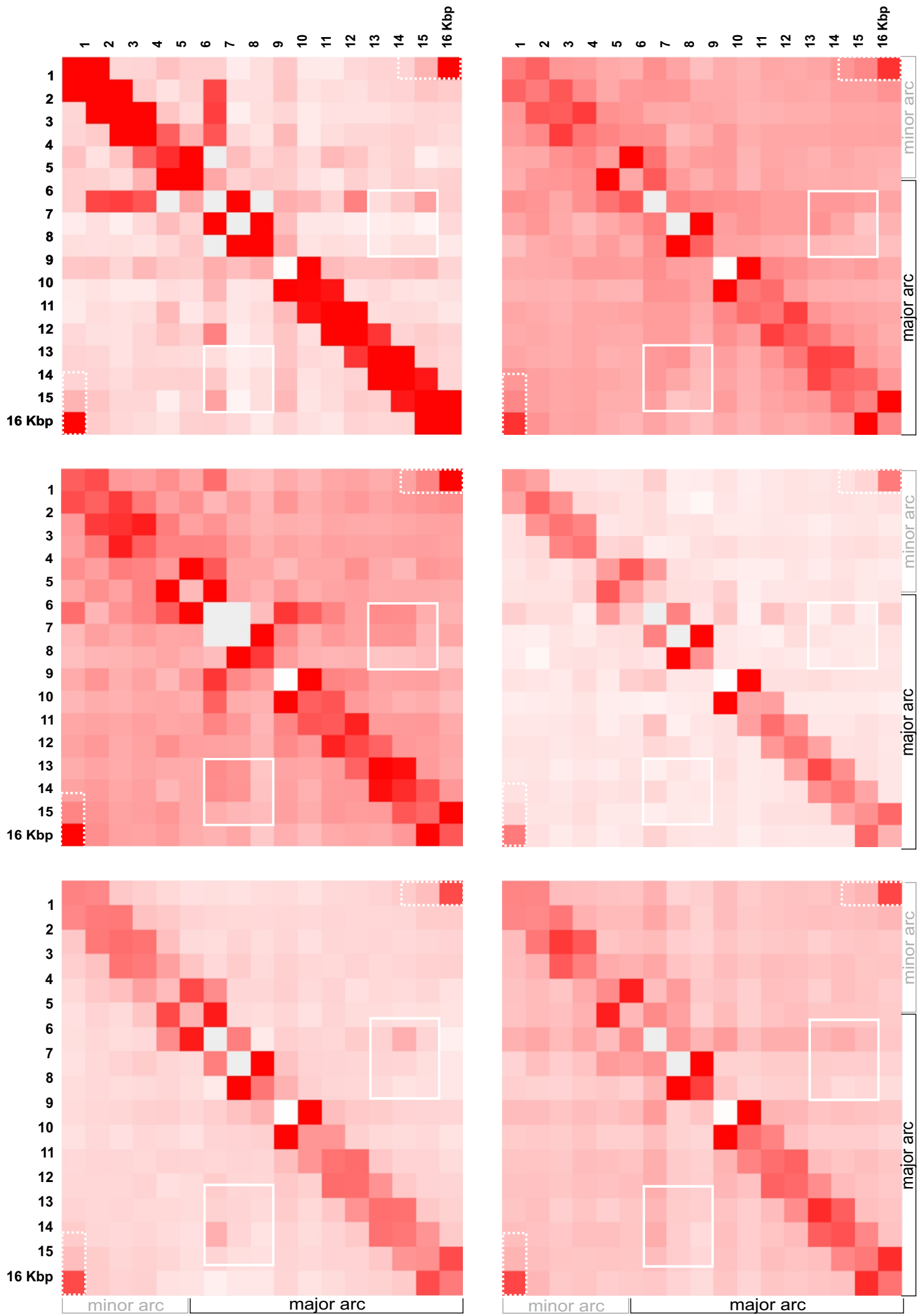

Supplement: Supplementary file 4 — Additional file 4. Hi-C contact matrix of mtDNA obtained from the human olfactory epithelium autopsies. The top row represents two contact matrixes from covid patients, middle and bottom rows represent the contact matrices from controls. Solid white squares mark the potential contact zone. Dotted white rectangles mark the contacts, emphasizing the circularity of AQmtDNA. [file 12915_2023_1606_MOESM4_ESM.pdf]
